# Comparison of Patient-Reported Outcome Measures Between Robotic-Assisted and Manual Total Hip Arthroplasty: A Systematic Review with a Minimum 2-Year Follow-Up

**DOI:** 10.3390/jcm14176036

**Published:** 2025-08-26

**Authors:** Itay Ron, Jacob Shapira, Ady H. Kahana-Rojkind, Roger Quesada, Benjamin G. Domb

**Affiliations:** 1 The Ruth and Bruce Rappaport Faculty of Medicine, Technion-Israel Institute of Technology, Haifa 3109601, Israel; jackob.sh@gmail.com; 2 Orthopedic Department, Rambam Medical Center, Haifa 3525408, Israel; 3 American Hip Institute Research Foundation, Chicago, IL 60018, USA; ady.kahana@americanhipinstitute.org (A.H.K.-R.); bendombpersonal@drdomb.com (B.G.D.)

**Keywords:** total hip arthroplasty, robotic hip arthroplasty, manual hip arthroplasty

## Abstract

**Background/Objectives:** Since robotic THA is a relatively new procedure, there is a paucity of high-quality research evaluating long-term PROMs, and as such this study aimed to compare the long term outcomes in robotic and manual THA. To systematically review the literature comparing mid- to long-term patient-reported outcome measures (PROMs) between robotic-assisted and manual THA. **Methods:** This study focused specifically on full-body text of studies comparing robotic and manual THA and comparing PROMs with a minimum of 2 years follow-up. Inclusion criteria were studies comparing robotic THA and manual THA and showing at least 1 PROMs with a minimum follow-up period of 2 years. **Results:** Five studies reported higher scores in 2-year follow-up for patient undergone robotic surgery. In addition, most studies did not show significant difference in dislocation nor revision rate between robotic and manual THA. Six studies assessed limb-length discrepancy using radiographic measurements and found no evidence of superior outcomes in either group. **Conclusions:** Based on the current evidence, this review identified evidence suggesting a trend toward better PROMs in patients operated on robotically. However, there was not enough evidence to conclude a correlation between lower risks for post-operative complications, revisions, and dislocations and robotic surgery.

## 1. Introduction

Total hip arthroplasty (THA) is a highly successful procedure for treating hip osteoarthritis. It is considered as one of the best elective procedures, improving daily functioning and patient quality of life [1,2]. The first robotic-assisted hip replacement was performed in 1992 using the ROBODOC system, and since then, innovation in this field have not stopped, resulting in a surge of publications showing early outcomes of robotic-assisted hip arthroplasty [3,4]. One of the main focuses of current research on robotic-assisted THA has primarily concentrated on component positioning, with evidence suggesting that robotic THA improves the accuracy of implant positioning [5,6]. In recent years, the application of robotic technology has emerged as an answer to reduce dislocations and complications. PROMs are considered the true objective reflection of patients’ postoperative well-being; however, even though studies have investigated this subject, there is still no consensus on the effect of robotics on postoperative outcomes following THA. As an elective procedure, its main purpose is to improve patients’ quality of life, and hence defining its effect on patients’ satisfaction and their effect on PROMs is crucial [7]. Due to the high costs of using robotic systems, and the fact that manual THA is already a proven successful procedure, this study aimed to compare the long term outcomes in robotic and manual THA [8,9]. Due to the fact that robotic THA is a relatively new procedure, there is a paucity of high-quality research evaluating long-term PROMs. In light of the rapid technological evolution and increasing adoption of robotic-assisted total hip arthroplasty (THA), several new studies have been published since the most recent systematic review, and therefore we deemed it necessary to conduct an updated systematic review to reassess the current evidence and address the emerging data published in recent years. The purpose of this study was to assess mid- to long-term patient-reported outcomes measures of patients post robotic-assisted THA. In attempt to clarify this aspect, this study systematically reviewed relevant literature comparing PROMs of patients who undergone robotic-assisted THA and manual THA. This study focused specifically on full-body text of studies comparing robotic and manual THA and comparing PROMs with a minimum of 2 years follow-up.

## 2. Materials and Methods

**Figure 1:**
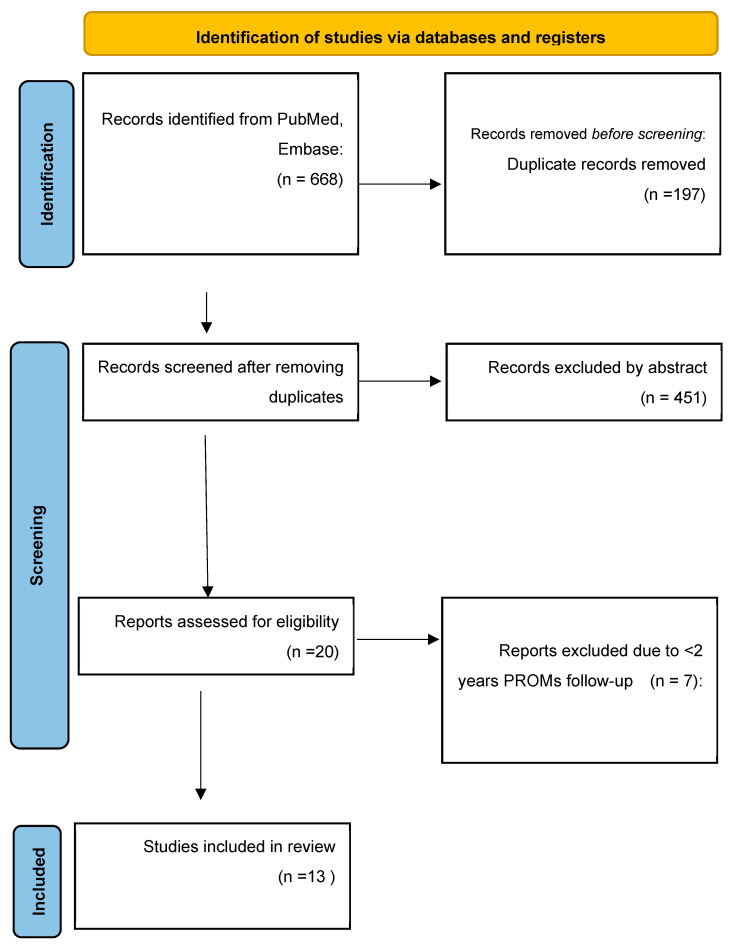
Identification of studies.

This study was conducted in accordance with the PRISMA (Preferred Reporting Items for Systematic reviews and Meta-Analyses) guidelines (Figure 1) [10]. Databases searched included PubMed (MEDLINE) and Embase. Keywords included in the literature search were “arthroplasty”, “hip replacement”, and “robotic”. Corresponding MeSH terms were “Arthroplasty, Replacement, Hip”; “Robotics”; “Arthroplasty”. Research was carried out by combining the aforementioned keywords and by using the relevant MeSH term for total hip replacement in PubMed. There were no restrictions on the publication date of the included studies. The literature search was conducted in November 2023; therefore, the most recent studies included correspond to those available at the time of the search. Inclusion criteria were studies comparing robotic THA and manual THA and showing at least 1 PROMs with a minimum follow-up period of 2 years. Studies were excluded if they were case reports or review articles, used cadavers, or if articles were published in language other than English. Surgical approach was not a restriction for inclusion, and studies utilizing both direct anterior and posterior approach were included. Subgroups such as revision THA, developmental dysplasia of the hip, or post-traumatic osteoarthritis could not be consistently identified across the included studies and therefore were not explicitly included or excluded.

Two independent reviewers manually screened titles and abstracts from all identified articles for eligibility. Systematic review was performed a priori in November 2023 with the International Prospective Register of Systematic Reviews (PROSPERO), registration CRD42023493090.

### 2.1. Quality Assessment

The quality of each of the included articles was assessed using the Risk of Bias in Non-Randomized Studies of Interventions (ROBINS-I) [11]. For randomized studies, the Cochrane Risk of Bias 2.0 (RoB 2.0) tool was used [12]. Any differences in scoring was discussed between the two reviewers, and a consensus was reached. The level of evidence of each study was determined using the standard criteria by Hohmann et al. [13]. Two studies included patients from the same database but from different time periods [14,15].

### 2.2. Data Extraction

Data documented from each of the studies included the title, author, publication date, study design, robotic system utilized, surgical approach, follow-up duration, demographics, study period, number of robotic THA and manual THA surgeries, pre-op and minimum 2-year follow-up PROMs, dislocations, revision surgeries and complications. All extracted data was organized in Microsoft Excel sheet.

### 2.3. Patient-Reported Outcome Measures (PROMs)

Patient-reported outcome measures (PROMs) were extracted from each included study, as reported by the original authors. The PROMs assessed encompassed a wide range of validated tools commonly used in hip arthroplasty research. These included the Harris Hip Score (HHS), Merle d’Aubigné Score, Western Ontario and McMaster Universities Osteoarthritis Index (WOMAC), Oxford Hip Score (OHS), Japanese Orthopaedic Association Score (JOA), and the University of California, Los Angeles Activity Score (UCLA) for functional assessment. Pain and satisfaction were evaluated using the Visual Analog Scale (VAS), Numeric Rating Scale (NRS), Harris Pain Score, and overall patient satisfaction ratings. General health-related quality of life was assessed using the 12-Item Short Form Survey (SF-12), Veterans RAND 12-Item Health Survey (VR-12), and the Health Status Questionnaire (HSQ), including its subdomains such as pain, physical functioning, and role limitations. In more recent studies, the Forgotten Joint Score (FJS/FJS-12) was also utilized to assess joint awareness during daily activities, with some analyses stratified by surgical approach.

## 3. Results

### 3.1. Study Identification and Quality

The initial search in PubMed (MEDLINE) and Embase yielded 668 articles (Figure 1). After removing duplicates, 471 articles were left for initial screening of titles and abstracts for relevancy. After first assessment, 20 articles were identified for a full-text review. Following full-text review, 13 articles met the inclusion criteria and were included. Seven studies were level III, retrospective cohort studies [14,15,16,17,18,19,20], four studies were prospective studies at level II [21,22,23,24], and two studies were level I RCTs [25,26]. Assessment using ROBINS-I tool for non-randomized trials showed a low-moderate risk of bias for the non-randomized trials due to their retrospective and observational nature. Using RoB 2.0 tool for randomized trials raised some concerns for risk of bias due to the difficulty of proper randomization and blinding of surgical interventions (Table 1 and Table 2). Further, only three studies included adequate description of surgeon experience using the robotic system [17,19,22].

### 3.2. Study Characteristics

This systematic review included in the analysis 13 studies which comprised a total of 2422 THA surgeries, among which 820 (33.9%) were robotic-assisted surgeries and 1602 (66.1%) manual THA. Male patients were operated on in 978 (40.4%) THA, and 1444 (59.6%) surgeries were for female patients. Six of the studies reviewed robotic THA using the ROBODOC (THINK Surgical, Inc., Fremont, CA, USA) robotic system, which assists in the preparation of femoral canal and stem, and seven studies focused on robotic THA using the Mako Robotic-Arm (MAKO Surgical Corp., Weston, FL, USA, [Stryker]) for acetabular reaming and cup placement. Furthermore, ROBODOC studies were older studies; all studies included in this review since 2018 focused on the Mako system only. Study and patient characteristics are shown in Table 3 and Table 4.

### 3.3. Patient Reported Outcomes Measures

Fifteen different PROMs were used in the 13 reviewed articles in this systematic review. The most common PROM was Harris Hip Score (HHS), which was used in eight different articles. Forgotten Joint Score (FJS) and WOMAC score were the second most used PROMs, as they appeared in four different articles. Out of the 13 articles reviewed, 5 articles reported better outcomes for robotic THA after at least 2-year follow-up, represented by at least 1 statistically significant PROMs in 2-year follow-up assessment, while the other 6 articles did not find statistical difference. Nishihara et al. [24] described better outcomes for robotic THA at 2-year follow-up by reporting higher Merle d’Aubigne score (*p* < 0.05). Bargar et al. [20] described higher scores for robotic THA in HSQ pain, Harris pain score, and WOMAC (*p* = 0.019; *p* = 0.025; *p* = 0.034, respectively). In their study, Domb et al. [14] showed four different PROMs with statistical significance at 5-year follow-up (HHS, FJS-12, VR-12, SF-12). Also, Perets et al. [15] described higher HHS and FJS scores for robotic surgery in a minimum 2-year follow-up. In contrast, Singh et al. [19] reported statistically higher FJS-12 scores at 2-year follow-up for patients who underwent manual THA compared with patients in the navigation and robotic cohorts (*p* = 0.004). Only nine studies also reported pre-op PROMs; all of them were not statistically significant [16,17,18,21,22,23,24,25,26]. All other PROMs are shown in Table 5.

### 3.4. Complications

All but one study in this review did not include any report regarding post-surgical complications, dislocations, or revision [16]. Three studies using the ROBODOC robotic system reported on intra-operative femoral fractures during manual THA. The only study which showed a statistical significance in this regard was Nishihara et al. [24]. which reported five intra-operative femoral fractures in the hand rasping group; all of these cases were in female patients.

Regarding dislocation rate, only four studies reported this subject. Honl et al. [25] reported about 11 (18%) cases of dislocation in the robotic THA group, while only 3 (4%) patients dislocated their hip in the manual hand rasping group, a significant difference (*p* < 0.001). Domb et al. [14] reported only one patient dislocating in the robotic THA group. Two studies reported zero dislocations in their study [18,22].

Regarding revisions, Honl et al. [25] presented higher rates for revision in robotic THA in their study. Two (3%) patients treated with manual implantation underwent revision surgery due to infection, while nine (15%) patients treated with robotic implantation had revision surgery (*p* = 0.007). Furthermore, rate of reoperation for a reason other than infection was 15% in the group treated with robotic implantation and 0% in the group treated with manual implantation. Bargar et al. [20] did not show statistical significance and reported one revision of the femoral component in each group for post-operative peri-prosthetic fracture, and three revisions and five revisions in the robotic and manual groups due to polyethylene wear, respectively. Perets et al. [15] reported one revision in the robotic-assisted group and three revisions in the manual group with no statistical significance, and Singh et al. [19] also reported no statistical significant difference in 90-day all-cause revisions. Summary of further complication in Table 6.

### 3.5. Leg-Length Discrepancy

Leg-length discrepancy was evaluated in post-operative radiographs in six studies [14,15,17,25,26]. Honl et al. [25] reported significantly less inequality and less variance in patients treated with robotic implantation (*p* < 0.001). Perets et al. [15] described similar outcomes as the robotic group had significantly less discrepancy. Ma et al. [17] showed significant differences in leg-length discrepancy only when comparing robotic THA and manual THA through a posterior approach; when robotics were compared to the direct anterior approach, there was no statistical significance. As opposed to that, three studies [14,18,26] reported their post-operative radiographic limb-length measurements and did not show a statistically significant discrepancy between the two surgical methods.

### 3.6. Operative Times

Five studies included information regarding operative times between robotic THA and manual THA [18,19,24,25,26]. Four studies showed statistically significant differences with longer operative times when using the robotic systems [19,24,25,26]. Nishihara et al. [24] showed a mean of 122 min for robotic THA and mean of 102 min for manual THA (*p* < 0.001). Honl et al. [25] showed a mean of 107.1 ± 29.1 min for robotic THA and a mean of 82.4 ± 23.4 for manual THA (*p* < 0.001). Lim et al. [26] recorded a mean of 103 min and 78 min for robotic and manual THA, respectively (*p* = 0.012). Singh et al. [19] reported 119.61 ± 36.36 min in the robotic THA group and 95.35 ± 60.96 min in the manual THA group (*p* < 0.001). Furthermore, Chai et al. [18] described shorter operative times for manual THA but with no statistically significant difference.

## 4. Discussion

This systematic review reviewed 13 articles which compared different outcomes of robotic THA and manual THA. In an era which values patients’ satisfaction and improved functionality, this study attempted to shed some light on the question whether robotic THA result in better and long term patient satisfaction by reviewing 2-year follow-up PROMs reported in the current literature. Of all the 13 studies included in this review, 5 [14,15,19,20,24] studies reported higher scores at 2-year follow-up for patients who had undergone robotic surgery. In addition, most studies did not show significant differences in dislocation nor revision rate between robotic and manual THA. Furthermore, six studies evaluated limb-length discrepancy through radiographic measurements and concluded no superior results for either of the groups.

There is already a consensus regarding the ability of robotic measures to achieve better accuracy and precision in restoring hip biomechanics and acetabular cup positioning when compared to manual THA [4,9,27]. Nonetheless, in recent years, there has been an increased focus on placing patients at the center of health care. To address the question of whether robotic-assisted THA is more beneficial for patients compared to manual THA, this review has focused specifically on PROMs. A recent systematic review by Sweet et al. [28] that aimed to investigate this subject was published in 2021 and reviewed seven articles comparing 2-year follow-up PROMs between robotic and manual THA. Their study concluded that since the technology of robotic THA is evolving and changing all the time, there is a paucity of high-quality evidence of this subject. In their review, two out of seven studies showed long-term PROMs favoring robotic THA, reporting on two different robotic systems [14,20]. In this systematic review, five [14,15,19,20,24] studies reported significantly better PROMs for robotic-assisted THA. These results may suggest an advantage in long-term PROMs with robotic-assisted THA. Correspondingly, the experience of using robotic systems for THA has increased, and therefore more studies point to proven results regarding PROMs. Although a consistent trend favoring robotic-assisted total hip arthroplasty (THA) in terms of improved patient-reported outcome measures (PROMs) is observed across the included studies, it is important to acknowledge that most of these studies did not report the Minimal Clinically Important Difference (MCID). Furthermore, different studies use different PROMs. Therefore, future studies should aim to incorporate MCID thresholds when evaluating PROMs in order to provide more robust and patient-centered evidence regarding the value of robotic-assisted THA.

Regarding leg-length discrepancy, differences in limb length are a common problem following THA and result in inferior outcomes and patient dissatisfaction, being the leading cause of litigation brought against orthopedic surgeons [29]. Thus, a critical obligation of robotic-assisted THA is maintenance or improvement of leg-length quality [30]. In this review, three studies [15,17,25] reported superior results in terms of leg-length discrepancy for robotic THA. Furthermore, a previous study by Nakamura et al. [31] showed less variance in post-operative limb-length in the robotic-milling group. These results corroborate the already-known consensus regarding the benefits of robotic systems for higher precision and accuracy in surgery [32].

In terms of complications, in this review, only Honl et al. [25] reported higher rates of revisions and dislocations in robotic-assisted THA. These results should be taken under careful consideration as this study was published 20 years ago and focused only on the ROBODOC system. Recent studies have presented promising results; Illgen et al. reported lower dislocation rate and similar infection rate for MAKO procedures compared to manual THA [33]. Furthermore, another study by Domb et al. reported only 1 technical complication in 50 cases performed with robotic assistance [34]. These results support the hypothesis that as time passes, the development of technology and surgical experience will improve and lead to better results in robotic surgeries, and this may even be backed up by corresponding research.

The introduction of new techniques and technologies in THA also contains some drawbacks. Out of five [18,19,24,25,26] studies reporting on operative times in this review, four [19,24,25,26] have shown statistical differences with shorter operative times for manual THA. Also, the fifth study described a trend of shorter operative times for manual THA. Much of this difference in operative times may be attributable to the learning curve as surgeons become accustomed to using robotic system [35,36]. Furthermore, longer operative times have been associated with increased expenses and risk of surgical site infection; these downsides must be considered when justifying the use of robotic THA [37]. It is likely that the longer operative times reported in earlier studies reflect, at least in part, the learning curve associated with the adoption of robotic systems. While detailed data on surgeon experience were not consistently available, this factor should be considered when interpreting differences in operative efficiency between robotic and manual THA.

### 4.1. Limitations

This systematic review had some limitations. As robotic-assisted THA is an evolving technology, most of the studies included in this review were retrospective and observational in their nature, revealing some of the reported outcomes with bias concerns; however, as far as we know, this systematic review is the most comprehensive review of this topic in the current literature, containing the highest number of articles included. Further, two RCT studies which were included in this review were published a long time ago and reported about results using the ROBODOC system only. Nevertheless, there was a heterogeneity in the studies included in terms of study design, surgery indication, outcome measures, surgical approach, surgeon experience, and follow-up period. Moreover, this study reviewed studies reporting both the ROBODOC system and the Mako system, as the two different robotic systems might have an effect on the generalizability of this study’s conclusions. It is important to note that the studies included in this review did not report Minimal Clinically Important Difference (MCID) values for the PROMs assessed. Since MCID is a key metric for interpreting the clinical relevance of patient-reported outcomes, its absence may limit the ability to draw firm conclusions regarding the true impact of robotic versus manual THA from the patient’s perspective. Furthermore, due to heterogeneity in study design, follow-up duration, and variability in how PROMs were reported, a formal meta-analysis and quantitative synthesis (e.g., forest plots) could not be performed, which limits the ability to statistically summarize the overall effect.

### 4.2. Conclusions

In conclusion, based on the current evidence, this review reported a trend towards better PROMs in patients operated on robotically. Furthermore, there was not enough evidence to conclude a correlation between lower risks for post-operative complications, revisions, and dislocations and robotic surgery. We hope that as this technology continues evolving, high-quality evidence will accumulate, shedding some more light on this exciting and fascinating technology.

## Figures and Tables

**Table 1:** Summary of quality assessment for non-RCT (ROBINS-I).

Study	Bias Due to Confounding	Bias in Selection of Participants	Bias in Measurement of Intervention	Bias Due to Deviations from Intended Intervention	Bias Due to Missing Data	Bias in Measurement of Outcomes	Bias in Selection of the Reported Result	Overall Risk of Bias
**Nishihara 2006** [24]	Moderate	Low	Moderate	Low	Low	Moderate	Moderate	Moderate
**Hananouchi 2007** [21]	Moderate	Low	Low	Low	Low	Moderate	Moderate	Moderate
**Bargar 2018** [20]	Moderate	Low	Low	Low	Moderate	Moderate	Moderate	Moderate
**Nakamura 2018** [23]	Serious	Low	Low	Low	Low	Moderate	Moderate	Serious
**Banchetti 2018** [16]	Moderate	Low	Low	Low	Moderate	Moderate	Moderate	Moderate
**Domb 2020** [14]	Low	Low	Low	Low	Low	Low	Low	Low
**Perets 2021** [15]	Low	Low	Low	Low	Low	Low	Low	Low
**Singh 2021** [19]	Low	Low	Low	Low	Low	Low	Low	Low
**Chai 2022** [18]	Low	Low	Low	Low	Low	Low	Low	Low
**Ma 2023** [17]	Moderate	Low	Low	Low	Low	Moderate	Moderate	Moderate
**Fontalis 2023** [22]	Moderate	Low	Low	Low	Moderate	Moderate	Moderate	Moderate

**Table 2:** Summary of quality assessment for RCT (RoB 2.0).

Study	Randomization Process	Deviations from Intended Intervention	Missing Outcome Data	Measurement of the Outcome	Selection of the Reporter Result	Overall Risk of Bias
**Honl 2003** [25]	Low	Some Concerns	Low	Some Concerns	Low	Some Concerns
**Lim 2015** [26]	Low	Low	Low	Some Concerns	Low	Some Concerns

**Table 3:** Characteristics of the included studies.

Study	Country	Level of Evidence	Study Design	Robot Used	Robotic Component Placed	Surgical Approach	Sample Size	
							Robotic THA	Manual THA
**Honl 2003** [25]	Germany	I	RCT	ROBODOC	Femoral Stem	Anterolateral	61	80
**Nishihara 2006** [24]	Japan	II	Prospective	ROBODOC	Femoral Stem	Postero-lateral	78	78
**Hananouchi 2007** [21]	Japan	II	Prospective	ROBODOC	Femoral Stem	No report	31	27
**Lim 2015** [26]	South Korea	I	RCT	ROBODOC	Femoral Stem	No report	24	25
**Bargar 2018** [20]	United States	III	Retrospective from 2 previous RCT cohorts	ROBODOC	Femoral Stem	Postero-lateral	45	22
**Nakamura 2018** [23]	Japan	II	Prospective	ROBODOC	Femoral Stem	No report	64	64
**Banchetti 2018** [16]	Italy	III	Retrospective	Mako	Acetabular Cup	Postero-lateral	56	51
**Domb 2020** [14]	United States	III	Retrospective	Mako	Acetabular Cup	Direct anterior and Posterior	66	66
**Perets 2021** [15]	United States	III	Retrospective	Mako	Acetabular Cup	Direct anterior and Posterior	85	85
**Singh 2021** [19]	United States	III	Retrospective	Mako	Acetabular Cup	Direct anterior and Posterior	135	929
**Chai 2022** [18]	China	III	Retrospective	Mako	Acetabular Cup	Postero-lateral	27	27
**Ma 2023** [17]	China	III	Retrospective	Mako	Acetabular Cup	Robotic Posterior approach, Manual Direct Anterior	40	40
						Posterior Approach Robotic, Manual	58	58
**Fontalis 2023** [22]	United Kingdom	II	Prospective	Mako	Acetabular Cup	Posterior	50	50

**Table 4:** Patient characteristics.

**Study**		**Age**		**Male**		**Female**	
		**Robotic THA**	**Manual THA**	**Robotic THA**	**Manual THA**	**Robotic THA**	**Manual THA**
**Honl 2003** [25]		71.5 ± 7.1	70.7 ± 8.3	24	24	37	56
**Nishihara 2006** [24]		58	58	14	14	64	64
**Hananouchi 2007** [21]		56.7 ± 9.2	57.4 ± 7.1	0	0	31	27
**Lim 2015** [26]		51.2 (19–67)	45.6 (21–65)	11	13	13	12
**Bargar 2018** [20]		59.1 (8.2)	59.8 (9.4)	35	12	10	10
**Nakamura 2018** [23]		57 ± 9	57 ± 9	12	11	52	53
**Banchetti 2018** [16]		71.5 ± 7.1	70.7 ± 8.3	31	26	25	25
**Domb 2020** [14]		59.01 ± 8.16	57.77 ± 10.50	24	25	42	41
**Perets 2021** [15]		57.0 ± 9.1	56.6 ± 9.6	37	37	48	48
**Singh 2021** [19]		61.62 ± 13.04	63.74 ± 10.04	59	401	76	528
**Chai 2022** [18]		43.04 ± 8.92	44.56 ± 9.53	0	0	27	27
**Ma 2023** [17]	Robotic vs. DAA	50.21 ± 10.89 (31–69)	50.26 ± 9.35 (27–69)	23	26	17	14
	Robotic vs. Posterior	51.15 ± 10. 96 (31–69)	51.88 ± 8.90 (29–72)	35	36	23	22
**Fontalis 2023** [22]		67 (50–77)	69 (49–80)	23	25	27	25

The values are given as the mean and SD or mean and range in parentheses.

**Table 5:** Outcome measures.

Study	Outcome Measures		Robotic THA	Manual THA	*p* Value
**Honl 2003** [25]	Merle d’Aubigne	Pre-op	9.7 ± 2.1	10.1 ± 1.9	0.37
		2-year	15.7 ± 2.2	14.9 ± 2.1	0.06
	Mayo	Pre-op	27.7 ± 15.6	28.1 ± 11.5	0.39
		2-year	73.1 ± 7.3	65.5 ± 9.1	0.07
	HHS	Pre-op	44.4 ± 12.9	47.6 ± 11.5	0.87
		2-year	85.9 ± 12.0	83.6 ± 11.9	0.06
**Nishihara 2006** [24]	Merle d’Aubigne	Pre-op	10.1 [6–14]	9.8 [5–16]	0.48
		2-year	17.4 [14–18]	17.1 [14–18]	**<0.05**
**Hananouchi 2007** [21]	Merle d’Aubigne	Pre-op	9.5 ± 2.7	9.9 ± 2.3	0.67
		2-year	17.8 ± 0.6	17.7 ± 0.7	0.83
**Lim 2015** [26]	HHS	Pre-op	52 [37–61]	55 [41–60]	0.155
		2-year	93 [85–100]	95 [89–100]	0.512
	WOMAC	Pre-op	60 [44–85]	61 [45–89]	0.517
		2-year	11 [6–17]	12 [5–15]	0.301
**Bargar 2018** [20]	Vas pain		4.69 ± 10.15	6.42 ± 10.89	0.112
	HSQ pain		83.75 ± 20.4	72.65 ± 16.31	**0.019**
	HSQ role physical		81.39 ± 28.25	70.88 ± 35.23	0.317
	HSQ physical functioning		84.26 ± 26.71	75.49 ± 26.43	0.102
	Total HSQ 12		683.52 ± 113.09	637.13 ± 104.53	0.087
	Harris pain score		41.81 ± 5.05	39.09 ± 7.37	**0.025**
	Total Harris		93.49 ± 8.77	89.5 ± 12.03	0.089
	WOMAC		8.44 ± 11.48	11.32 ± 11.92	**0.034**
	UCLA		6.09 ± 1.86	5.71 ± 1.45	0.087
**Nakamura 2018** [23]	JOA	Pre-op	48 ± 11	52 ± 15	0.07
		10-year	97 ± 5	96 ± 7	0.159
**Banchetti 2018** [16]	HHS	Pre-op	44.3 ± 8.1	46 ± 8.7	0.43
		2-year	85.6 ± 8.1	85.15 ± 7.7	0.72
	WOMAC	Pre-op	70.1 ± 14.8	68.9 ± 11.2	0.62
		2-year	6.8 ± 11.1	6.9 ± 10.2	0.95
	NRS	Pre-op	8.6 ± 1.2	8 ± 1.1	0.084
		2-year	0.82 ± 1.5	0.84 ± 1.5	0.9377
**Domb 2020** [14]	HHS		90.57 ± 13.46	84.62 ± 14.45	**<0.001**
	FJS-12		82.69 ± 21.53	70.61 ± 26.74	**0.002**
	VAS		1.27 ± 2.20	1.07 ± 1.87	0.45
	Satisfaction		8.91 ± 2.00	8.52 ± 2.62	0.35
	VR-12 Mental		60.76 ± 5.94	58.97 ± 6.93	0.17
	VR-12 Physical		50.30 ± 8.83	45.92 ± 9.44	**0.002**
	SF-12 Mental		56.59 ± 5.60	56.20 ± 6.62	0.81
	SF-12 Physical		48.97 ± 9.21	44.01 ± 10.26	**0.001**
**Perets 2021** [15]	HHS		91.0 ± 12.4	84.4 ± 14.9	***p* < 0.001**
	FJS		80.2 ± 21.3	68.6 ± 27.3	***p* = 0.003**
	VAS		9.0 ± 1.9	8.9 ± 1.9	0.591
**Singh 2021** [19]	FJS-12		73.35 ± 25.33	74.63 ± 25.96	**0.004**
	FJS-12 posterior approach sub-analysis		73.35 ± 25.33	71.51 ± 28.21	0.022
**Chai 2022** [18]	HHS	Pre-op	63.0 ± 13.0	58.4 ± 13.6	0.269
		2-year	94.5 ± 3.3	93.5 ± 3.9	0.313
	WOMAC		13.4 ± 7.4	15.1 ± 11.5	0.512
**MA 2023** [17]	HHS (Robotic vs. DAA)	Pre-op	55.98 ± 11.41	48.29 ± 19.81	0.081
		2-year	87.04 ± 7.06	85.33 ± 8.34	0.202
	HHS (Robotic vs. Posterior Approach)	Pre-op	51.56 ± 13.99	49.71 ± 21.80	0.708
		2-year	89.38 ± 6.81	85.33 ± 8.81	0.019
**Fontalis 2023** [22]	OHS (Oxford Hip Score)	Pre-op	22.6 ± 8.8	21 ± 7	0.312
		3-year	42 [37–43.25]	41 [37.5–43]	0.914
	UCLA	Pre-op	4 [3–5]	4 [3–4]	0.994
		3-year	7.5 [6–9]	7 [6–8]	0.381
	FJS (Forgotten Joint Score)	Pre-op	52.3 ± 9.5	53.8 ± 8.8	0.41
		3-year	89 [82.75–92]	86 [80–89]	0.065

The values are given as the mean and SD or mean and range in parentheses. Follow-up time is 2 years, unless otherwise described.

**Table 6:** Complications.

Study	Complications		Dislocations	Revision	Limb Length Discrepancy
	Robotic THA	Manual THA			
**Honl 2003** [25]	Nerve palsy, 4 (7%). Prolonged wound healing, 4 (7%). DVT, 3 (5%). Heterotopic ossification, 8 (10%).	Nerve palsy (1%). Prolonged wound healing, 3 (4%). DVT, 3 (4%). Heterotopic ossification, 6 (10%).	rTHA, 11 (18%) mTHA, 3 (***p* < 0.001**)	Infection: mTHA, 2 (3%) rTHA, 9 (15%) (*p* = 0.007) Non-infection: mTHA 0 rTHA 9 (15%) (***p* < 0.001**)	rTHA resulted in significantly less inequality and variance. (***p* < 0.001**)
**Nishihara 2006** [24]	No Complications	Intraoperative femoral fractures, 5 **(Statistically significant)**	Not reported	Not reported	Not reported
**Hananouchi 2007** [21]	Heterotopic ossification, 1 patient.	Intraoperative femoral fractures, 2	Not reported	No revisions	Not reported
**Lim 2015** [26]	No complications	Intraoperative femoral fractures, 2	Not reported	Not reported	mTHA, 2 LLD outliers
**Bargar 2018** [20]	No complications	No complications	No dislocations	mTHA, 6 rTHA, 4 (post-operative periprosthetic fracture; polyethylene wear)	Not reported
**Nakamura 2018** [23]	Heterotopic ossification, 19 (30%)	Heterotopic ossification, 12 (19%)	Not reported	No revisions	Not reported
**Banchetti 2018** [16]	Not reported	Not reported	Not reported	Not reported	Not reported
**Domb 2020** [14]	Superficial infections, 2. Deep vein thrombosis, 1.	Minor numbness in the thigh, 3. Sciatic nerve injury, 1.	rTHA, 1	No significance	No significance
**Perets 2021** [15]	Superficial infections, 6. DVT, 1.	Superficial infection, 2. lateral femoral cutaneous nerve numbness, 2. Numbness around the incision scar, 1. Calcar split, 1.	Not reported	rTHA, 1 (1.1%) mTHA, 3 (3.5%)	Significantly less discrepancy in the robotic group
**Singh 2021** [19]	Not reported	Not reported	Not reported	No significance 90-day all-cause revisions	Not reported
**Chai 2022** [18]	No complications	Wound exudation, 2.	No dislocations	Not reported	No significance
**MA 2023** [17]	Not reported	Not reported	Not reported	Not reported	Significant differences in postoperative LLD between rTHA and mTHA posterior approach.
**Fontalis 2023** [22]	Not reported	Not reported	No dislocations	No revision surgeries	Not reported
